# Technical Validation and Clinical Implications of Ultrasensitive PCR Approaches for *EGFR*-Thr790Met Mutation Detection in Pretreatment FFPE Samples and in Liquid Biopsies from Non-Small Cell Lung Cancer Patients

**DOI:** 10.3390/ijms23158526

**Published:** 2022-07-31

**Authors:** Javier Simarro, Gema Pérez-Simó, Nuria Mancheño, Emilio Ansotegui, Carlos Francisco Muñoz-Núñez, José Gómez-Codina, Óscar Juan, Sarai Palanca

**Affiliations:** 1 Molecular Biology Unit, Service of Clinical Analysis, Hospital Universitario y Politécnico La Fe, 46026 Valencia, Spain; javier_simarro@iislafe.es (J.S.); gema_perez@iislafe.es (G.P.-S.); 2 Clinical and Translational Cancer Research Group, Instituto de Investigación Sanitaria La Fe (IIS La Fe), 46026 Valencia, Spain; 3 Pathology Department, Hospital Universitario y Politécnico La Fe, 46026 Valencia, Spain; manchenyo_nur@gva.es; 4 Pulmonology Department, Hospital Universitario y Politécnico La Fe, 46026 Valencia, Spain; ansotegui_emi@gva.es; 5 Radiology Department, Hospital Universitario y Politécnico La Fe, 46026 Valencia, Spain; carlos.munoznunez@gmail.com; 6 Medical Oncology Department, Hospital Universitario y Politécnico La Fe, 46026 Valencia, Spain; gomez_joscod@gva.es (J.G.-C.); juan_osc@gva.es (Ó.J.); 7 Biochemistry and Molecular Biology Department, Universidad de Valencia, 46010 Valencia, Spain

**Keywords:** non-small cell lung cancer, molecular biology, *EGFR* p.(Thr790Met) mutation, ultrasensitive assays, prognostic and predictive biomarkers

## Abstract

In pretreatment tumor samples of *EGFR*-mutated non-small cell lung cancer (NSCLC) patients, *EGFR*-Thr790Met mutation has been detected in a variable prevalence by different ultrasensitive assays with controversial prognostic value. Furthermore, its detection in liquid biopsy (LB) samples remains challenging, being hampered by the shortage of circulating tumor DNA (ctDNA). Here, we describe the technical validation and clinical implications of a real-time PCR with peptide nucleic acid (PNA-Clamp) and digital droplet PCR (ddPCR) for *EGFR*-Thr790Met detection in diagnosis FFPE samples and in LB. Limit of blank (LOB) and limit of detection (LOD) were established by analyzing negative and low variant allele frequency (VAF) FFPE and LB specimens. In a cohort of 78 FFPE samples, both techniques showed an overall agreement (OA) of 94.20%. *EGFR*-Thr790Met was detected in 26.47% of cases and was associated with better progression-free survival (PFS) (16.83 ± 7.76 vs. 11.47 ± 1.83 months; *p* = 0.047). In LB, ddPCR was implemented in routine diagnostics under UNE-EN ISO 15189:2013 accreditation, increasing the detection rate of 32.43% by conventional methods up to 45.95%. During follow-up, ddPCR detected *EGFR*-Thr790Met up to 7 months before radiological progression. Extensively validated ultrasensitive assays might decipher the utility of pretreatment *EGFR*-Thr790Met and improve its detection rate in LB studies, even anticipating radiological progression.

## 1. Introduction

*EGFR* activating mutations define a specific molecular subset of non-small cell lung cancer (NSCLC) patients. In-frame deletions in exon 19 and the point mutation p.(Leu858Arg) in exon 21 constitute approximately 90% of all *EGFR* activating mutations found in NSCLC [1]. These mutations confer sensitivity to treatment with tyrosine kinase inhibitors (EGFR-TKIs) [2]. Despite the high response rate, the vast majority of patients will develop resistance to EGFR-TKI treatment and experience disease progression. In patients treated with first- or second-generation EGFR-TKIs, the most common resistance mechanism is the *EGFR* point mutation c.2369C > T; p.(Thr790Met) in exon 20, which is detected in 50–60% of patients with progressive disease [3].

The mechanism by which this mutation emerges remains unclear, although two hypotheses have been proposed [4]. The acquisition model suggests that *EGFR*-Thr790Met mutation emerges during EGFR-TKI treatment [5]. However, *EGFR*-Thr790Met mutation is detected by conventional methods in 1–8% of EGFR-TKI-naïve patients which suggests a selection model of a minor clone harboring this mutation [6,7]. 

Tumor heterogeneity is a well-known event in cancer with critical impact in diagnosis and cancer treatment [8,9]. The pretreatment presence of tumor cells clones harboring *EGFR*-Thr790Met mutation may have been underestimated because of the limit of detection (LOD) of the conventional *EGFR* testing methods. These methods, currently based on real-time PCR, such as Taqman PCR or Scorpion Amplification Refractory Mutation System (SARMS), reach a LOD of 1–5% mutant alleles [10]. New ultrasensitive approaches are able to identify specific mutations with a LOD even down to 0.01%, but also require more technical training, experience and validation efforts to obtain reproducible and transferable results. Matrix-assisted laser desorption ionization–time of flight mass spectrometry (MALDI-TOF MS), mutant allele enrichment PCR techniques (PNA-LNA, PCR Clamp, etc.), COLD-PCR methods or digital PCR approaches have been developed for ultrasensitive mutation detection in FFPE samples [11,12,13,14]. However, using these techniques, a detection rate of *EGFR*-Thr790Met ranging from 17% to 80% has been reported and, as a consequence, the emergence model and its clinical impact remains unclear [15,16,17,18,19,20,21,22].

Nevertheless, in patients with progressive disease with first- or second-generation EGFR-TKIs, *EGFR*-Thr790Met is a well-established predictive biomarker of osimertinib treatment benefit [23]. Since obtaining a second tumor biopsy for molecular testing is not feasible in the majority of patients, liquid biopsy (LB) constitutes a non-invasive approach to analyze circulating tumor DNA (ctDNA) in blood and other body fluids. However, due to the low amount of ctDNA in peripheral blood, current evidence shows that LB studies show suboptimal sensitivity, with tumor-detected and ctDNA-undetected cases being relatively common [24].

Ultrasensitive approaches such as digital droplet PCR (ddPCR) have shown promising results as a new diagnostic strategy able to increase the detection rate of *EGFR*-Thr790Met in LB studies [25]. However, due to the relevance of its detection, validation studies are needed before implementing this technique to routine molecular diagnosis. Moreover, monitoring tumor mutations through serially obtained liquid biopsies is a highly promising approach for patients’ clinical management. These studies have demonstrated the ability to detect *EGFR*-Thr790Met before radiological progression [26,27] and its use as a new standard of tumor progression is currently being researched [28].

In this work, we report the in-house validation of two ultrasensitive PCR methods for pretreatment *EGFR*-Thr790Met detection in order to establish the prevalence of this mutation and to assess its prognostic value. Moreover, we also report the diagnostic performance of ddPCR for *EGFR*-Thr790Met detection in LB at treatment failure and during EGFR-TKI treatment follow-up.

## 2. Results

### 2.1. Dilution Bank Preparation

**Figure 1:**
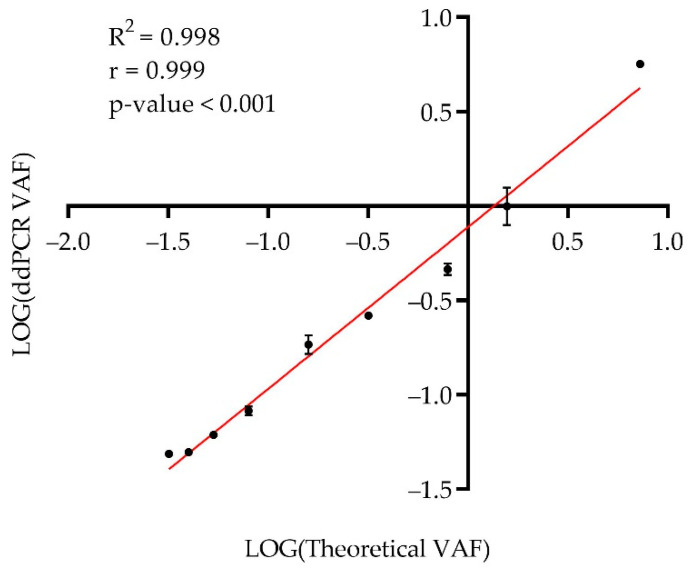
Correlation between theoretical VAF (%) and observed VAF (%) by ddPCR. Observed values are the mean of two independent experiments with error bars representing the standard deviation. Logarithmic transformation of both variables was applied to enhance visualization.

DNA from the H1975 cell line and DNA from healthy donors were processed by ddPCR in three independent experiments to detect the *EGFR*-Thr790Met mutation before preparing the dilution bank. H1975 was found to harbor this mutation at a VAF of 80.50 ± 0.63% while DNA from healthy donors was confirmed as negative. A dilution bank with an increasing quantity of wild type DNA was prepared as described in Section 4. These aliquots were tested in two independent experiments to ensure the VAF. The observed VAF showed a high and significant correlation with the theoretical VAFs (R^2^ = 0.998, Pearson correlation test; r = 0.999 and *p*-value ˂ 0.01) Figure 1. 

### 2.2. Limit of Blank (LOB) and Limit of Detection (LOD) Establishment

The study of wild type FFPE samples established the LOB of the PNA Clamp TaqMan assay as 0.0364% while the LOB of the ddPCR assay was set to 0.0957%. Low-positivity samples were included in the validation procedure. We established a LOD of 0.0996% for PNA Clamp TaqMan assay and 0.1336% for ddPCR assay. For both assays, between LOB and LOD values, we established an “uncertainty zone” in which we cannot truly assess *EGFR*-Thr790Met presence. Regarding ddPCR validation in LB samples, LOB was established in 1.6 mutant copies/mL of plasma and LOD was set at 3.0 copies/mL of plasma.

### 2.3. PNA Clamp TaqMan Assay and ddPCR Results: Comparison

**Figure 2:**
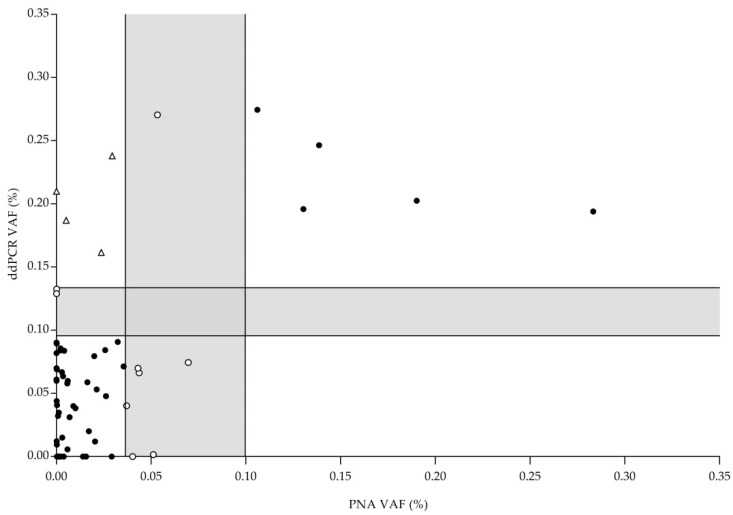
ddPCR and PNA Clamp TaqMan assays results for 78 FFPE specimens. Each symbol represents one sample whose position is determined by PNA Clamp TaqMan assay result (*X*-axis) and ddPCR assay result (*Y*-axis). Gray bars represent uncertainty zone of each methodology which is limited by LOB and LOD values. (●) Samples with concordant results, (○) samples in uncertainty area and (∆) samples with discordant results. Positive samples with VAF higher than 0.3% are not depicted.

A total of 78 FFPE diagnostic samples were studied by ddPCR and PNA Clamp assays. In PNA Clamp TaqMan assays, seven samples showed a VAF between LOB and LOD (uncertain result) (7/78; 8.97%) while in ddPCR assays, two different samples gave an uncertain result (2/78; 2.56%). Consequently, excluding these samples, a total of 69 samples gave a reliable result by both methodologies (69/78; 88.46%). In both approaches, 18 samples were positive while 47 were classified as not detected. Only four samples were positive in the ddPCR assay and not detectable with PNA Clamp TaqMan assay (overall agreement, OA: 94.20% (Figure 2). These data suggest that both methods are useful for the detection of *EGFR*-Thr790Met mutation in FFPE samples. 

### 2.4. EGFR-Thr790Met Prevalence in Pretreated Stage IV NSCLC Association with Patients’ Characteristics

Thirty-four stage IV NSCLC patients with *EGFR*-Thr790Met genotype determined by ultrasensitive methods were selected to assess the relationship between this mutation and patients’ clinical–pathological characteristics. Nine patients (9/34; 26.47%) showed *EGFR*-Thr790Met pretreatment while the other 25 did not show this mutation (25/34; 73.53%). No statistically significant associations were found between pretreatment *EGFR*-Thr790Met mutation detection and clinical–pathological characteristics of patients. However, *EGFR*-Thr790Met was more frequent in male patients (66.7% vs. 33.3%), in patients without bone metastasis (88.9% vs. 11.1%) and in older patients (77.8% vs. 22.2%). Clinicopathological characteristics of patients according to *EGFR*-Thr790Met genotype are shown in Table 1.

### 2.5. EGFR-Thr790Met Pretreatment Status and Progression-Free Survival (PFS)

**Figure 3:**
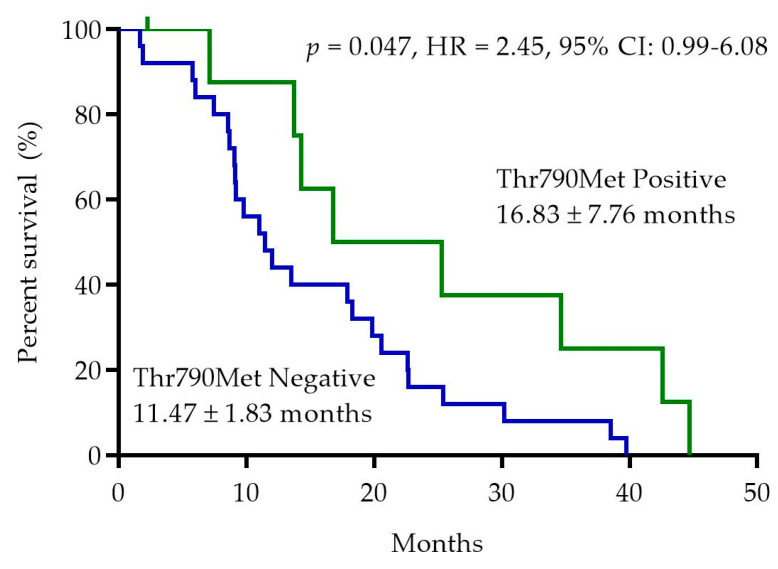
Duration of progression-free survival according to consensus *EGFR*-Thr790Met pretreatment status determined by PNA Clamp TaqMan Assay and ddPCR. HR: Hazard ratio, CI: Confidence interval.

At the time of the analysis, all patients progressed on first-line treatment with EGFR-TKIs (one patient censored). Median PFS was significantly higher in the *EGFR*-Thr790Met-positive patients (16.83 ± 7.76 months) than in negative patients (11.47 ± 1.83 months) (*p* = 0.047; HR 2.45, 95% CI: 0.99–6.08) (Figure 3). 

### 2.6. EGFR-Thr790Met Detection in LB at the Time of Disease Progression

Among the patients recruited in the LB cohort, we detected *EGFR*-Thr790Met in 12 samples (12/37; 32.43%) using Cobas^®^ EGFR Mutation Test v2 (CE-IVD). However, using ddPCR we were able to detect this mutation in five additional samples (17/37; 45.95%). ddPCR showed a sensitivity of 100%, a specificity of 80% and an OA of 86.49%. The five discordant samples showed an average of 27.40 (range: 5.02–86.11) mutant copies/mL of plasma. Mutant alleles were below the LOD of the Cobas^®^ assay (100 mutant copies/mL of plasma) in all discordant samples.

In two of the 20 *EGFR*-Thr790Met-negative patients, LB studies were expanded to other body fluids and ddPCR assay was able to detect *EGFR*-Thr790Met in pleural and cerebrospinal fluid, respectively. 

**Figure 4:**
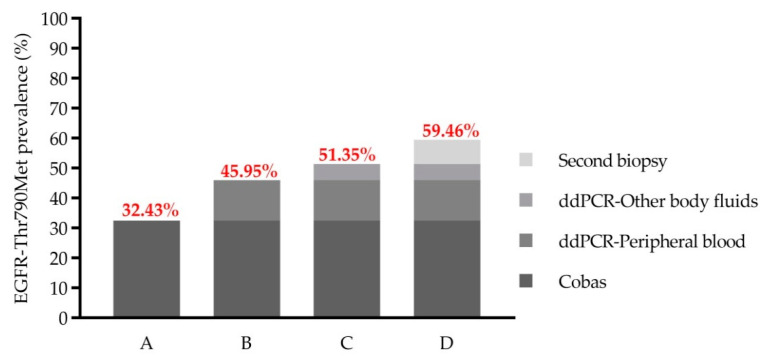
Increase in EGFR-Thr790Met prevalence at the time of disease progression according to different testing strategies. A: Cobas^®^, B: ddPCR—peripheral blood, C: ddPCR—peripheral blood + ddPCR—other body fluids and D: Strategy C + second tissue biopsies. Prevalence obtained with each strategy is depicted.

Among the remaining 18 patients, nine underwent bronchoscopy to obtain a second tumor biopsy. Molecular studies were performed in seven patients (no malignant cells were detected in two biopsies), with *EGFR*-Thr790Met detected in three cases by conventional methods. Taken together, these results show that the global *EGFR*-Thr790Met resistance mechanism’s prevalence in our cohort was 22/37 (59.46%) (Figure 4). 

Regarding clinical implications of *EGFR*-Thr790Met, all 22 patients showing *EGFR*-Thr790Met started osimertinib as a second-line treatment with a response rate of 18/20 (90.00%; two patients with incomplete clinical data). Interestingly, in the two patients with progressive disease with osimertinib, *EGFR*-Thr790Met was detected by both the Cobas^®^ and ddPCR method in plasma obtained from peripheral blood.

### 2.7. Early Detection of EGFR-Thr790Met in LB by ddPCR during Treatment Follow-Up

**Figure 5:**
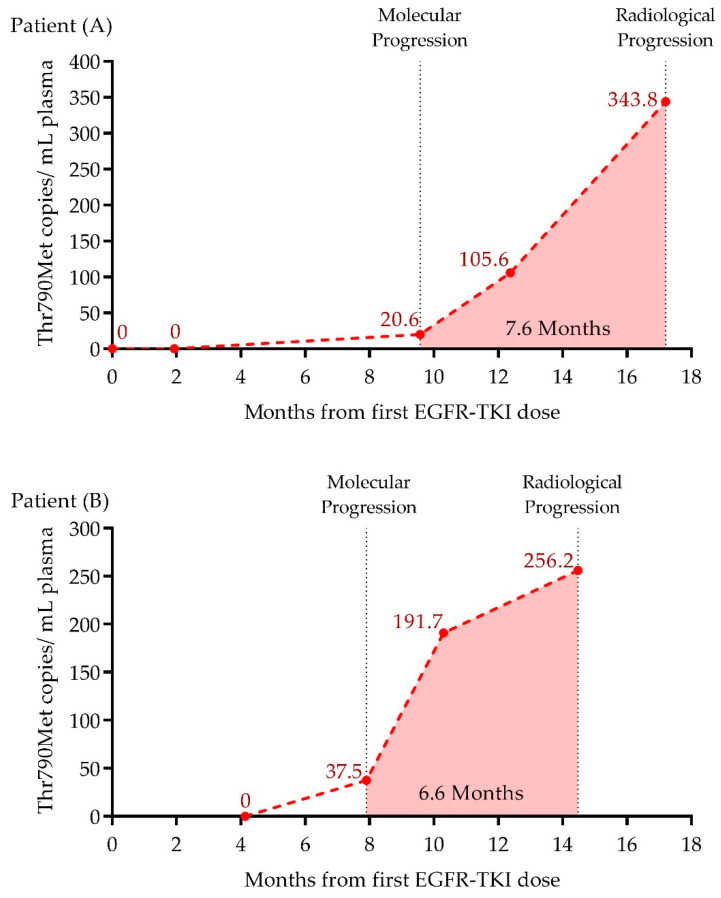
Dynamics of *EGFR*-Thr790Met mutation detected by ddPCR during follow-up in NSCLC patients treated with EGFR-TKI. Patients A and B (**A**,**B**). Red dots represent the *EGFR*-Thr790Met copies/mL of plasma detected in each sample. Molecular progression and radiological progression are depicted. Light red filled area comprises the months between these moments.

Serially obtained liquid biopsies during EGFR-TKI treatment of two patients with *EGFR*-Thr790Met detected in progressive disease were prospectively analyzed by ddPCR. In both cases, ddPCR was able to anticipate radiological progression by detecting 20.0 and 37.5 mutant copies/mL of plasma 7.6 and 6.6 months in advance, respectively. An increase in plasma *EGFR*-Thr790Met abundance was observed three months later, that reached its maximum at radiological progression with 343.0 and 256.0 mutant copies/mL of plasma for patients A and B, respectively (Figure 5).

## 3. Discussion

*EGFR*-mutated NSCLC patients benefit from targeted therapy with EGFR-TKI. Acquisition and selection models have been proposed to explain the emergence of *EGFR*-Thr790Met resistance mutation, however, no consensus has been reached due to methodological challenges and, as a consequence, its pretreatment clinical utility remains unknown. In contrast, this mutation plays a crucial role in progression, since positive patients are eligible for a second-line targeted treatment with osimertinib. In this scenario, LB is positioned as a non-invasive alternative to second tissue biopsy studies for molecular characterization of progressive disease. Implementation of ultrasensitive assays in clinical routine LB studies could identify patients with circulating *EGFR*-Thr790Met alleles in low abundance, minimizing second biopsies and even anticipating radiological progression.

Using ddPCR and a PNA Clamp TaqMan assay, we have detected the presence of pretreatment *EGFR*-Thr790Met mutation in 26.5% of stage IV NSCLC patients. In the literature, several groups have reported variable detection rates due to the series of patients included, the diversity of ultrasensitive assays and the different approaches developed for their technical validation. 

Regarding ddPCR, Watanabe et al. and Vendrell et al. detected EGFR-Thr790Met mutation in 79.9% and 66.0% of pretreatment FFPE cases, respectively [16,17]. In contrast, Beau-Faller et al. and Matsumoto et al. reported a lower detection rate using ddPCR (8% and 42.4%, respectively) [29,30]. Interestingly, Tatematsu et al. reported a detection rate of 40% in a group of 20 frozen tumor samples, and although there was a limited number of patients, they suggested that the study of non-FFPE samples may have reduced its false positive rate [22]. In this sense, Lettig et al. established a detection rate of 17% in a cohort of 114 patients using a ddPCR assay, detecting an increase in false positive droplets when processing DNA from FFPE samples [15]. Moreover, Rosell et al. and Costa et al. reported a detection rate of 35.0% and 65.3%, respectively, using a PNA Clamp assay previously validated with DNA from cell lines [20,21].

The variability in the reported frequency of pretreatment *EGFR*-Thr790Met suggests that its detection may be compromised by the quality of FFPE DNA. Fixation and paraffin-embedding processes produce a highly fragmented and chemically modified DNA that can lead to artificial mutation calls in ultrasensitive assays because of the low LOD of these techniques [31,32].

For this reason, in this work we focused on the implementation and validation of two ultrasensitive assays for *EGFR*-Thr790Met testing in FFPE samples. First of all, we decided to use DNA obtained from FFPE samples and commercial FFPE-like samples in order to establish the LOB and LOD. In these samples, DNA quantity and quality were as compromised as in clinical samples, making the established LOB and LOD more precise and transferable. Moreover, the requirement of agreement between ddPCR and PNA Clamp TaqMan assay constitutes a stricter genotyping strategy than in most of the published studies. Consequently, the prevalence of *EGFR*-Thr790Met mutation established in our work is lower than in most of the previously reported studies using ultrasensitive methods [16,17,21,30,33].

The clinical significance of the pretreatment low frequency of *EGFR*-Thr790Met mutation has not been determined. Our findings are consistent with Fujita et al. who reported a longer PFS in *EGFR*-Thr790Met-positive patients (10 vs. 8 months, *p* = 0.44) [33] and also with Vendrell et al. and Lettig et al. who have described a better prognosis in pretreatment *EGFR*-Thr790Met-positive patients (29.2 vs. 11 months, *p* = 0.009 and HR = 0.40, *p* = 0.04, respectively) [15,16]. However, several studies reported a significantly shorter PFS in pretreatment positive patients (Su et al., 6.7 vs. 10.2 months *p* < 0.05; Lee et al., 6.3 vs. 11.5 months *p* < 0.001; Rosell et al., 12 vs. 18 months *p* = 0.05; Costa et al., 9.8 vs. 15.8 months *p* = 0.0185; Maheswaran et al., 7.7 vs. 11.5 months *p* < 0.001 and Matsumoto et al., 6.9 vs. 13.8 months *p* < 0.001) [18,19,20,21,30,34]. This observation is also supported by Ma et al. and Ding et al. who concluded in two meta-analyses that *EGFR*-Thr790Met confers a worse prognosis in EGFR-TKI-naïve patients (HR = 2.21 and HR = 1.95, respectively) [35,36]. Moreover Beau-Faller et al. recently reported a significantly shorter PFS only in patients harboring this mutation in a VAF > 1%, suggesting that abundance of this resistant clone could also influence its prognostic value [29].

Interestingly, Chmielecki et al. reported in a preclinical study a slower growth of cultured tumor cells harboring *EGFR*-Thr790Met mutation [37]. This observation is reflected in clinical studies which describe an indolent progression and better outcome in patients who develop *EGFR*-Thr790Met mutation at disease progression [38,39], possibly because other resistance mechanisms involve more complex genetic changes [40]. However, heterogeneity of *EGFR* mutant tumors may include minor clones harboring diverse resistance mechanisms, leading to a complex evolutionary model of TKI-resistant clones [41].

The heterogeneity of the samples analyzed and the different methods employed to detect this mutation in previously reported studies prevent us drawing solid conclusions. Extensively validated and standardized ultrasensitive studies are needed to establish a consensus about pretreatment *EGFR*-Thr790Met prevalence and its clinical impact. Moreover, since the approval of osimertinib as a first-line treatment in *EGFR*-mutated NSCLC patients, pretreatment *EGFR*-Thr790Met arouses great interest. Its detection could lead to the application of a more personalized medicine in this subgroup of patients [42].

Regarding LB studies, ctDNA quantity varies between patients and over time, being influenced by tumor location, treatment and cell proliferation rates [43]. For this reason, ultrasensitive approaches such as ddPCR are extremely promising. Cobas^®^ EGFR Mutation Test v2 is a CE-IVD marked method for detecting EGFR mutations in NSCLC patients with a LOD of 100 mutant copies/mL of plasma for *EGFR*-Thr790Met mutation. However, in our work, the ddPCR assay with a LOD of 3 mutant copies/mL of plasma led to the detection of *EGFR*-Thr790Met in 13.51% of positive patients apart from those detected with Cobas. The technical validation of ddPCR for *EGFR*-Thr790Met detection in LB samples reported in this work led to its implementation in routine diagnostics under UNE-EN ISO 15189:2013 accreditation.

Moreover, ddPCR studies have allowed the detection of *EGFR*-Thr790Met prior to radiological progression. Although longitudinal monitoring in LB is not included in clinical guidelines, the clinical utility of early *EGFR*-Thr790Met detection (molecular progression) arouses great interest as an early radiological progression marker [28].

In conclusion, the determination of *EGFR*-Thr790Met by ultrasensitive assays in pretreatment FFPE biopsies is feasible only with extensive validation studies that ensure the correct genotyping of NSCLC patients. Our work reveals the presence of pretreatment *EGFR*-Thr790Met in 26.5% of stage IV NSCLC patients. However, the clinical significance of pretreatment *EGFR*-Thr790Met mutation remains unresolved and needs to be assessed in a larger cohort. Moreover, we report that implementation and technical validation of ddPCR studies for *EGFR*-Thr790Met detection in LB are able to identify a higher number of positive patients and even anticipate radiological progression.

## 4. Materials and Methods

### 4.1. Patients

**Figure 6:**
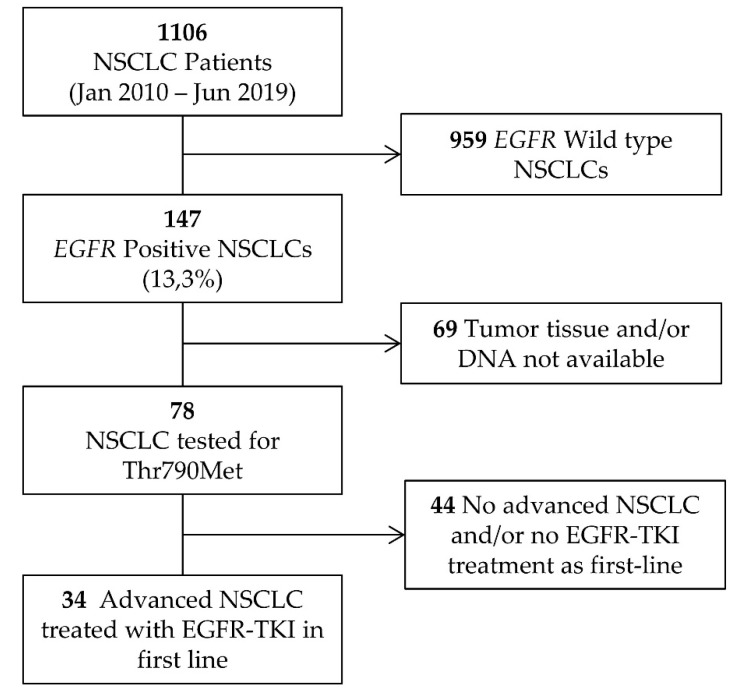
Flow chart showing the patients included in the pretreatment cohort. NSCLC: Non-small cell lung cancer. EGFR-TKI: Epidermal growth factor receptor tyrosine kinase inhibitor.

One thousand one hundred and six patients were recruited via the Medical Oncology Department at the Hospital Universitario y Politécnico La Fe in Valencia (Spain) from January 2010 to September 2019. One hundred and forty-seven patients had NSCLC positive for activating *EGFR* mutations (13.3%). Only 78 of 147 patients had sufficient formalin fixed paraffin embedded tissue (FFPE) for the ultrasensitive *EGFR*-Thr790Met mutation testing. Of these, 34 met the eligibility criteria for inclusion in the pretreatment cohort: (1) diagnosed with advanced NSCLC and (2) treated in first line with first- or second-generation EGFR-TKI. A flow chart of patient enrollment is shown in Figure 6. The main characteristics of these patients are shown in Table 2.

In the liquid biopsy cohort, 37 *EGFR*-positive NSCLC patients were recruited from April 2016 to December 2019. Peripheral blood samples were obtained at radiological progression with first- or second-generation EGFR-TKI. Tissue samples were obtained for nine patients without EGFR-Thr790Met detectable in LB studies. Additionally, in two patients, six liquid biopsy samples obtained during first-line EGFR-TKI treatment were retrospectively analyzed for *EGFR*-Thr790Met detection.

All patients showed their agreement by signing the informed consent by the Health Department in accordance with the recommendations of the Declaration of Human Rights, the Conference of Helsinki and institutional regulations, and approved by the Hospital Ethics Committee.

### 4.2. Reference Materials

Wild type genomic DNA obtained from peripheral blood of healthy donors and genomic DNA from the H1975 cell line (*EGFR*-Thr790Met positive) were used for the standard curve preparation of the PNA Clamp TaqMan assay. For the validation procedure of both ultrasensitive assays in FFPE samples, we used wild type DNA from FFPE reference material obtained from Horizon Discovery (Waterbeach, UK) and FFPE samples previously characterized in-house. Multiplex I cfDNA Reference Standard Set (Horizon Discovery) containing wild type cfDNA and two aliquots of cfDNA harboring the *EGFR*-Thr790Met variant (0.1%, and 1%) were used in the validation procedure of ddPCR for LB assays.

### 4.3. DNA Extraction Plasma Isolation

All molecular analyses were carried out at the Molecular Biology Unit (UBM) of the Clinical Analysis Department, an ISO 15189-certified laboratory (*Entidad Nacional de Acreditación*, ENAC, Nº1302/LE2445). FFPE sections were macrodissected by a pathologist to select regions containing the highest proportion of tumor cells (≥30%). Genomic DNA (gDNA) was isolated from five 5 μm thick FFPE sections using Deparaffinization Solution and the GeneRead DNA FFPE Kit (Qiagen, Hilden, Germany) according to the manufacturer’s protocol. Peripheral blood was collected into two 8.5 mL Vacutainer PPT EDTA-K2 Gel separator tubes (BD Biosciences, Franklin Lakes, NJ, USA). Samples were centrifuged at 4 °C (1800 g) for 10 min. Supernatant was subsequently centrifuged at 4 °C (16,000 g) for 10 min to remove cell debris. cfDNA was isolated from 4 mL of cell-free plasma using a MagMAX Cell-Free DNA Isolation Kit (ThermoFisher Scientific, Waltham, MA, USA). gDNA from peripheral blood of healthy donors and from cell lines was isolated using an UltraClean™ Blood DNA Isolation Kit (MO-BIO, Carlsbad, CA, USA). In all samples, DNA concentration was assessed using a Qubit 3.0 fluorometer with the DNA HS (High Sensitivity) Assay Kit (ThermoFisher Scientific, Waltham, MA, USA).

### 4.4. EGFR Mutation Screening

*EGFR* mutation screening was routinely performed in FFPE and LB samples using Cobas^®^ EGFR Mutation Test v2 (CE-IVD) (Roche Diagnostics, Basel, Switzerland) following the manufacturer’s instructions.

### 4.5. PNA Clamp TaqMan Assay

The peptide nucleic acid (PNA) Clamp TaqMan assay was implemented as described by Costa et al. [21]. PCR reactions, using previously described primers and probes, were carried out in a 7500 Fast Real-Time PCR System and results were analyzed with the provided software (Applied Biosystems, Foster City, CA, USA). For each sample, we analyzed the cycle of threshold (CT) of the wild type allele (in the absence of PNA) and the CT of the mutant *EGFR*-Thr790Met allele (in the presence of PNA). The difference (ΔCT) between the CT of the mutant allele and the CT of the wild type allele is an estimate of the percentage of the mutated allele since there is a logarithmic relationship between ΔCt and the proportion of the mutated allele in the total allele population (variant allele frequency, VAF).

To establish the logarithmic relationship between ΔCt and VAF, we prepared a standard curve as previously described. We diluted DNA from the H1975 cell line into increasing concentrations of wild type donor DNA. This dilution bank consisted of 9 dilutions of a theoretical VAF ranging from 7.84% to 0.03%, which were tested in quadruplicate to obtain the ΔCt values. Using this standard curve, we were able to interpolate the ΔCt values of tested samples to obtain the VAF. In order to reduce inter-assay variability, in every run, we processed three standard curve dilutions to obtain a mean conversion factor as follows: (ΔCt value of dilution in standard curve experiment/ΔCt value in experiment). We used this conversion factor to multiply the ΔCt value obtained for each sample before interpolating.

### 4.6. Digital Droplet PCR (ddPCR)

*EGFR*-Thr790Met mutation testing by digital droplet PCR (ddPCR) was carried out with a QX200 Droplet Digital PCR System (Bio-Rad, Hercules, CA, USA) by using the ddPCR Mutation Assay VAL EGFR T790M (dHsaMDV2010019). ddPCR was performed in duplicate on 20 ng of gDNA obtained from FFPE samples or cell lines. LB samples were tested in quadruplicate using 9 µL of cfDNA. Droplets were generated with a QX200 Droplet Generator and PCR reaction was carried out in a C1000 Touch Thermal Cycler (Bio-Rad, Hercules, CA, USA). The cycling conditions for the PCR reaction included an initial incubation at 95 °C for 10 min, 40 cycles of 94 °C for 30 s and 55 °C for 60 s and enzyme inactivation at 98 °C for 10 min. After thermal cycling, the plates were transferred to a QX200 Droplet Reader (Bio-Rad, Hercules, CA, USA) for fluorescence reading. ddPCR data were analyzed with the QuantaSoft software (Bio-Rad, Hercules, CA, USA), detecting positive droplets for *EGFR*-Thr790Met mutant and wild type probes and then establishing the VAF of *EGFR*-Thr790Met mutation for FFPE sample testing and the *EGFR*-Thr790Met allele copies/mL of plasma for LB testing.

### 4.7. Limit of Blank (LOB) and Limit of Detection (LOD)

LOB and LOD were established for PNA Clamp TaqMan assay in FFPE samples and for ddPCR in both FFPE and LB samples [44]. The LOB was established as the highest mutant signal (VAF or mutant copies/mL of plasma) that could be detected in wild type samples: LOB = mean VAF + 1.645 × SD. Samples with low abundance of *EGFR*-Thr790Met were processed for LOD establishment as follows: LOD = LOB + 1.645 × SD_(low concentration sample)_.

To ensure that these limits were transferable to the tested samples, we used FFPE samples and cfDNA-like standards. For LOB establishment with FFPE samples, we analyzed seven FFPE *EGFR*-Thr790Met-negative samples previously characterized by next generation sequencing (NGS) (average read depth of 3500×) and 4 FFPE reference materials obtained from Horizon Discovery (Waterbeach, UK). Regarding LOD establishment, we studied five low *EGFR*-Thr790Met allele frequency samples (VAF = 0.100%, 0.150%, 0.200%, 0.220% and 0.230%). These samples were tested in triplicate and the mean of the SD obtained for each sample was used to calculate LOD. Regarding the LB validation procedure, for LOB establishment 10 wild type standards were processed and five standards with a VAF of 0.1% and 1%, respectively, were used for LOD establishment.

### 4.8. Statistical Analyses

Quantitative variables were summarized by their mean and standard deviation, and categorical variables by absolute and relative frequencies. Simple linear regression analysis and a Pearson correlation test were used to evaluate the relationship of theoretical VAF and that detected by ddPCR. Comparison among ddPCR results and PNA Clamp TaqMan assay results was made by determining overall agreement. Statistical association between the *EGFR*-Thr790Met genotype and qualitative variables was assessed by a chi-square test or the Fisher exact test. For PFS analyses, patients without radiological progression were censored. All time-to-event outcomes were estimated using the Kaplan–Meier method and compared across groups using log-rank testing (univariate analysis). A Cox proportional-hazards model was used to evaluate the association between the *EGFR*-Thr790Met genotype and the PFS of patients. Statistical analyses were carried out with the statistical package SPSS v.21 (IBM, Armonk, NY, USA) and GraphPad Prism Software version 7.0.2, (San Diego, CA, USA). *p*-values < 0.05 were considered statistically significant.

## Figures and Tables

**Table 1:** Patient’s characteristics according to the presence of *EGFR*-Thr790Met.

Characteristics	T790M Negative (*n* = 25) *n* (%)	T790M Positive (*n* = 9) *n* (%)	*p*-Value
**Sex**			0.139
Male	9 (36.0%)	6 (66.7%)	
Female	16 (64.0%)	3 (33.3%)	
**Age (years)**			0.240
˂65	13 (52.0%)	2 (22.2%)	
≥65	12 (48.0%)	7 (77.8%)	
**Smoking status**			0.254
Never smoker	17 (68.0%)	4 (44.4%)	
(Former) smoker	8 (32.0%)	5 (55.6%)	
**CNS metastasis**			1.000
No	18 (72.0%)	7 (77.8%)	
Yes	7 (28.0%)	2 (22.2%)	
**Bone metastasis**			0.214
No	15 (60.0%)	8 (88.9%)	
Yes	10 (40.0%)	1 (11.1%)	
Type of *EGFR* mutation *			1.000
Deletion exon 19	15 (62.5%)	5 (55.6%)	
Leu858Arg	9 (37.5%)	4 (44.4%)	
**EGFR-TKI Treatment**			0.216
Erlotinib	15 (60.0%)	3 (33.3%)	
Gefitinib	4 (16.0%)	4 (44.4%)	
Afatinib	3 (12.0%)	0 (0.0%)	
Dacomitinib	1 (4.0%)	0 (0.0%)	
Erlotinib–Gefitinib	1 (4.0%)	1 (11.1%)	
Erlotinib + Ramucirumab	1 (4.0%)	0 (0.0%)	
Erlotinib + Bevacizumab	0 (0.0%)	1 (11.1%)	

CNS: Central nervous system. *EGFR*: Epidermal growth factor receptor. * Patient with p.(Leu858Arg) and concomitant p.(Ser768Ile) was excluded from the analysis.

**Table 2:** Epidemiological and clinical–pathological characteristics of the patients included in the pretreatment cohort.

Characteristics	*n*	%
**Sex**		
Male	15	44.12%
Female	19	55.88%
**Age (years)**		
Mean	65.47	
Range	32–85	
**Smoking status**		
Never smoker	21	61.76%
Former smoker	6	17.65%
Current smoker	7	20.59%
**Histologic type**		
Adenocarcinoma	33	97.06%
Squamous	1	2.94%
**CNS metastasis**		
Yes	9	26.47%
No	25	73.53%
**Bone metastasis**		
Yes	11	32.35%
No	23	67.65%
**Type of *EGFR* mutation**		
Deletion 19	20	58.82%
Leu858Arg	13	38.24%
Leu858Arg/Ser768Ile	1	2.94%
**EGFR-TKI Treatment**		
Erlotinib	18	52.94%
Gefitinib	8	23.53%
Afatinib	3	8.82%
Dacomitinib	1	2.94%
Erlotinib–Gefitinib	2	5.88%
Erlotinib + Ramucirumab	1	2.94%
Erlotinib + Bevacizumab	1	2.94%

EGFR-TKI: Epidermal growth factor receptor tyrosine kinase inhibitor.
